# Cloning and Characterization of a *Phragmites australis* Phytochelatin Synthase (*PaPCS*) and Achieving Cd Tolerance in Tall Fescue

**DOI:** 10.1371/journal.pone.0103771

**Published:** 2014-08-18

**Authors:** Cuizhu Zhao, Jin Xu, Qiang Li, Shuo Li, Peng Wang, Fengning Xiang

**Affiliations:** 1 The Key Laboratory of Plant Cell Engineering and Germplasm Innovation, School of Life Sciences, Shandong University, Jinan, Shandong, China; 2 College of Agronomy, Northwest A&F University, Yangling, Shanxi, China; 3 Key Laboratory of Agricultural Water Resources, Center for Agricultural Resources Research, Institute of Genetics and Developmental Biology, Chinese Academy of Sciences, Shijiazhuang, China; University of Vigo, Spain

## Abstract

The production of phytochelatins (PCs) provides an important means for plants to achieve tolerance to cadmium (Cd) toxicity. A reed gene encoding PC synthase (*PaPCS*) was isolated and its function tested through its heterologous expression in a strain of yeast sensitive to Cd. Subsequently, the Cd sensitive and high biomass accumulating species tall fescue was transformed either with *PaPCS* or *PaGCS* (a glutamyl cysteine synthetase gene of reed) on their own (single transformants), or with both genes together in the same transgene cassette (double transformant). The single and double transformants showed greater Cd tolerance and accumulated more Cd and PC than wild type plants, and their Cd leaf/root ratio content was higher. The ranking in terms of Cd and PC content for the various transgenic lines was double transformants>*PaGCS* single transformants>*PaPCS* single transformants>wild type. Thus *PaGCS* appears to exert a greater influence than *PaPCS* over PC synthesis and Cd tolerance/accumulation. The double transformant has interesting potential for phytoremediation.

## Introduction

Heavy metal pollution caused by a combination of natural leaching and anthropogenic activity is becoming a significant environmental problem. Thousands of hectares of arable land have been contaminated in China, representing a significant health hazard to the population [1]–[3]. Phytoremediation is seen as a favorable strategy to remove the contamination. The optimal phytoremediating plant needs to be highly productive in terms of biomass and efficient in terms of accumulation of heavy metals [4], [5]. One such species is the reed *Phragmites australis* (Cav.) Trin. ex Steud, a most effective accumulator of Cd, Pb and Zn, and has been widely exploited as a sewage treatment wetland species [6]. However, the most efficient accumulators appear to be poor in terms of biomass production [7]–[9]. As a result, it has been proposed that an effective approach could be based on the engineering of heavy metal accumulation and tolerance into a species already recognized as being an effective biomass producer [10]–[12]. Tall fescue (*Festuca arundinacea* Schreb), a particularly vigorous grass species with broad climatic adaptation, could be an attractive recipient species, since turf grasses tolerate regular mowing, thus allowing for the ready disposal of the heavy metals translocated into their foliage. A number of genes involved in the uptake of and tolerance to heavy metals have been identified, and some have already been successfully transferred into plants [13]–[16], and it has been demonstrated that the heterologous expression of some of these improves the levels of heavy metal tolerance and accumulation.

The presence of heavy metals tends to induce the production of phytochelatins (PCs) in plants [17], [18]. The PCs form a family of oligopeptides able to neutralize the toxicity of heavy metals by chelation [19], [20]. Two key rate-limiting enzymes in the synthesis of PCs are phytochelatin synthase (PCS) and γ-glutamyl cysteine synthetase (γ-GCS) [21]. Their involvement in the accumulation of heavy metals has been experimentally confirmed in both *Arabidopsis thaliana* and *Brassica juncea* [22], [23] but it is unclear which of these two enzymes is the most important [24], [25]. We have previously shown that the γ-GCS glutamyl cysteine synthetase gene of *P. australis* (*PaGCS*) is a key component of the species' heavy metal tolerance, and that when this gene is expressed in the fast-growing species *Agrostis palustris*, tolerance to Cd toxicity is measurably enhanced [26]. In the present study we further explored the potential of *P. australis* as a donor of phytoremediation genes by testing the effect of expressing *PaPCS* and/or *PaGCS* in tall fescue.

## Materials and Methods

### Cloning of *Phragmites australis* phytochelatin synthase gene

Total RNA extracted from frozen *P. australis* leaf tissue using the TRIzol reagent (Invitrogen, Carlsbad, CA, USA) formed the template for the M-MLV reverse transcriptase driven synthesis of cDNA (TaKaRa Bio Group, Otsu, Japan). The resulting cDNA was amplified using the primer pair 5′-CTTCCAG(A/T)CTCA(G/A)TCGGAGC and 5′-ATTGC(G/C)ACTCCT(T/A)GACAGCA to obtain an initial *PaPCS* sequence. After sequencing this amplicon, further primers were derived to perform 3′-RACE and 5′-RACE (TaKaRa 3′- and 5′-Full RACE Core set), according to the manufacturer's protocols.

### Yeast complementation assay

*Sac*I/*Xba*I *PaPCS* cDNA fragments were sub-cloned into the pYES2 vector and transformed into *Saccharomyces cerevisiae* mutant strain YK44 (ura3-52 his3-200, *ΔZRCΔCot1*, mating type *α*) using the lithium acetate method [27]. Yeast cells harboring either an empty pYES2 vector or pYES2-PaPCS were cultured on SD-Ura medium (Difco BRL, USA) at 30°C until the OD_600_ reached 0.5. The cells were pelleted by centrifugation from a 500 µL aliquot of the culture and re-suspended in deionized water in 10^−1^, 10^−2^, and 10^−3^ concentrations, and then inoculated into 40 µL YPGAL (1% w/v yeast extract, 2% w/v peptone, 2% w/v galactose, Difco) medium in the presence of 100 µM CdCl_2_. All experiments were done in quadruplicate.

### Construction of plant expression vectors

The *Xba*I/*Sac*I *PaPCS* cDNA fragment was cloned into the pROKII vector (Fig. S1A), and the resulting pROK/PaPCS fusion was transformed into *E. coli* DH10B by thermal shock. The *35S::PaPCS-Tnos* fragment released from pROK/PaPCS and the equivalent for *PaGCS* [26] were fused and inserted into the plant gene expression vector pCAMBIA3301 based on NPTII as the selectable marker (Fig. S1B). The recombinant vector (p3301/PG) was then transformed into *E. coli* DH10B.

### Transformation of tall fescue

The binary plasmids pROK/PaGCS, pROK/PaPCS and p3301/PG were separately introduced into the *Agrobacterium tumefaciens* strain AGL1. Putative transformants were selected by including 50 mg/L kanamycin in the culture medium. Subsequent tissue culture and agroinfection of the hypocotyledonary axis were performed as reported by Fu et al (2007). Prior to agroinfection, *F. arundinacea* embryogenic calli were grown for one week on MS medium [28] containing 2 mg/L 2,4-dichlorophenoxyacetic acid (2,4-D). Following the agroinfection process, the embryogenic calli were exposed to 2 mg/L and 50 mg/L kanamycin for two weeks, after which the surviving calli were transferred to a differentiation medium (MS medium containing 1 mg/L 6-BA, 1 mg/L IAA and 50 mg/L kanamycin) for one month. Rooted seedlings were hardened on differentiation medium with the addition of 2 mg/L clobutrazol and 50 mg/L kanamycin for two weeks before being transplanted into soil in a greenhouse.

### Plant material and stress treatment

Transgenic and wild type (WT) *F. arundinacea* rooted seedlings were transplanted into soil in a greenhouse (22±3°C under a 16 h photoperiod with a photosynthetic photon flux density of 45 µmol m^−2^ s^−1^). The seedlings were at tillering stage after being transplanted into the soil for 30 days. Then, WT and transgenic tiller clones were transferred into Hoagland's solution, and exposed for five days to 150 µM CdCl_2_.

### Genetic analysis of putative transformants

Genomic DNA was isolated from the leaf of candidate transformants using the CTAB method [29]. A fragment beginning within the 35S promoter and ending within the *PaGCS* coding region was amplified as previously described [26] for the detection of *PaGCS* transformants. To detect candidate *PaPCS* transformants, a primer pair was designed to target a similar fragment of the *PaPCS* transgene (primer sequences were 5′-CTTCCAGACTCAGTCGGAGC and 5′-ATTGCGACTCCTTGACAGCA). Each 20 mL PCR reaction contained 0.4 µM of each primer, 400 µM dNTP, 1.5 mM MgCl_2_, 1.25 U rTaq DNA polymerase (Takara, Japan) and either 300 ng plant DNA or 15 pg plasmid. Each PCR consisted of a denaturation step (95°C/5 min), followed by 35 cycles of 95°C/40 s, 55°C/60 s 72°C/60 s, ending with an extension step of 72°C/10 min. PCR products were separated by 1% agarose gel electrophoresis. The presence of the transgene was also tested by Southern hybridization. For this purpose, 15 µg *Eco*RI- or *Sac*I digested genomic DNA was electrophoresed through a 0.8% agarose gel and electrophoretically transferred onto a Hybond N^+^ membrane (Amersham, Piscataway, NJ, USA). The membranes were hybridized with the *PaGCS* or *PaPCS* amplicons labeled with digoxigenin using the random primer method (DIG High Prime DNA Labeling and Detection Starter Kit II, Roche, Basel, Switzerland). Hybridization, washing and signal detection were performed according to the manufacturer's instructions.

### Biomass assay and the assessment of Cd^2+^ uptake

The biomass of WT and transgenic tiller clones exposed to 150 µM CdCl_2_ for five days was recorded. Each plant was thoroughly washed with distilled water, divided into leaves, shoots, and roots. Root samples were washed for 10 min at 4°C in 2 mM CaSO_4_ and 10 mM EDTA, and rinsed three times in water, and then placed into oven dried to a constant weight. A sample of 30 mg dry matter was digested in HNO3 and HClO4 (4∶1 v/v). Then it was evaporated to dryness and dissolved in 2 mol/L HNO_3_. The concentration of Cd^2+^ in the solvent was determined as described by Vitória, Lea & Azevedo [30], using an inductively coupled plasma mass spectrometer (ICP-MS). All experiments were done in quadruplicate.

### MDA content

Top most expanded leaves of transgenic and wild-type tall fescue with or without Cd treatment were collected. Malondialdehyde (MDA) content was determined by measuring the concentration of thiobarbituric acid-reacting material present, using thiobarbituric acid (TBA) [31]. Fresh leaves were homogenized with 2 mL of TBA reagent (15% w/v ti-ichloroacetic acid and 0.25 M HCl), and treated in a boiling water bath for 15 minutes. After cooling, the suspension was centrifuged at 10,000× g for 10 minutes. The supernatant was then separated. The absorbance was measured at 535 nm. The MDA concentration was determined by the specific absorbance coefficient (156 mM^−1^ cm^−1^). The MDA concentrations were measured after a 0 h, 6 h, 12 h, 24 h, 48 h, 72 h and 120 h exposure to 150 µM CdCl_2_. All treatments and experiments were using different plants done in quadruplicate. The MDA concentration unit was µmol·g^−1^DW.

### Enzyme activity measurement

Top most expanded leaves of transgenic and wild-type tall fescue with or without Cd treatment were collected. Peroxidase (POD) and superoxide dismutase (SOD) activity were measured as described by Scebba et al. [32]. Fresh leaves were immediately frozen in liquid nitrogen and then ground in liquid nitrogen. The powder was suspended in 0.5 mL 0.1 M Tris pH 8.0, 1 mM PMSF, 1% (w/v) polyvinylpyrrolidone, 1% (w/v) sodium ascorbate and 1% (v/v) β-mercaptoethanol. Extracts were centrifuged twice at 26,000 g for 20 minutes (4°C), and the clarified supernatant was used for determination of enzyme activity.

Total protein of the crude extract was measured using the method of Bradford [33]. Different amounts (0.005, 0.010, 0.020 and 0.040 mL) of crude extract were added to a reaction mixture. The mixture contained 50 mM sodium phosphate buffer pH 7.8, 0.1 mM ethylenediaminetetraacetic acid, 13 mM methionine, 2 µM riboflavine and 75 µM 3-(4,5)-dimethylthiazol-2-yl-2,5-diphenyl-tetrazolium bromide (MTT). Exposing the mixture to cool white fluorescent light for 15 minutes started the reaction. The light was then switched off, the tubes were stirred and the blue color was measured at 560 nm. POD (E.C.1.11.1.7) activity was assayed in a reaction mixture. The mixture contained 10 mM potassium phosphate buffer pH 7.0, 10 mM H_2_O_2_ solution, 20 mM guaiacol and 0.01 mL of crude extract. Adding H_2_O_2_ and guaiacol solution at the same time started the reaction. The activity was determined by monitoring the increase of absorbance at 470 nm, as a result of guaiacol oxidation. Both SOD and POD enzyme activities were measured after a 0 h, 6 h, 12 h, 24 h, 48 h, 72 h and 120 h exposure to 150 µM CdCl_2_. All treatments and experiments were using different plants done in quadruplicate. POD activity unit was Δ460·min^−1^·g^−1^. SOD activity unit was Unit·mg^−1^.

### The non-protein thiol, GSH and PCs quantification

Total glutathione (GSH) content was determined as described by Gupta et al. [34]. Frozen tillers (700 mg FW) was homogenized in 0.1 M sodium phosphate buffer (pH,8.0) and 25% HPO_3_, centrifuged at 20,000 g for 20 minutes and GSH was fluorometrically determined in the supernatant after 15 minutes incubation at 25°C with o phthaldehyde (OPT) and the fluorescent intensity was recorded at 420 nm after excitation at 350 nm on a Hitachi Fluorescence Spectrophotometer (Model No. 650-60). The non-protein thiol (TNP-SH) content was determined as described by Tu et al. [35]. The PC content were measured by HPLC as described by Gupta et al., using PC2 and PC3 as PC standards [34]. TNP-SH, GSH and PC content were measured after a 0 h, 6 h, 12 h, 24 h, 48 h, 72 h and 120 h exposure to 150 µM CdCl_2_. All treatments and experiments were using different plants done in quadruplicate. TNP-SH, GSH and PC content unit were µmol·g−1DW.

### Statistical analysis

For each statistical test, a threshold of *P*<0.05 was applied to indicate statistical significance. The data were analyzed using the SAS statistical package (v5.1, SAS Institute Inc., Cary, NC, USA).

## Results

### Isolation and function of *PaPCS*

**Figure 1:**
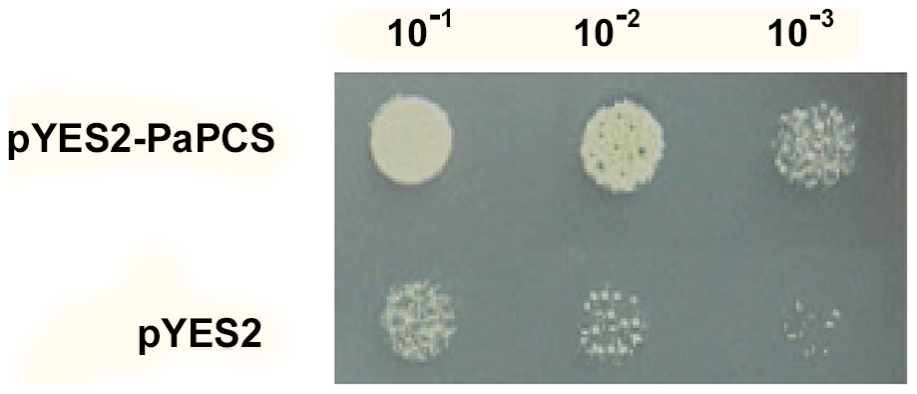
Functional testing of *PaPCS* in the Cd sensitive yeast strain YK44. Yeast cells harboring either an empty pYES2 vector or pYES2-PaPCS were cultured and inoculated into 40 µL YPGAL medium in 10^−1^, 10^−2^, and 10^−3^ concentrations in the presence of 100 µM CdCl_2_. After 48 h, the growth of transgenic cells heterologously expressing *PaPCS* was compared to control cells carrying an empty pYES2 vector. All experiments were done in quadruplicate.

The consensus regions shared by the PCS sequences present in bread wheat, pea, onion, *Zinnia elegans* and tomato provided the target for the design of PCR primers. A full-length *P. australis* cDNA was obtained by RACE-PCR. The *PaPCS* (GenBank accession number JX826285) sequence length was 1497 bp, and comprised a single open reading frame encoding a 498 residue polypeptide of predicted molecular mass ∼54.9 kDa. A phylogenetic analysis showed that its nucleotide sequence shared considerable similarity with its homologs from bread wheat (84.3%), bermudagrass (84.3%) and rice (70.9%). The closest related peptide sequence was the PCS from bermudagrass (Fig. S2). The presence of the *pYES2-PaPCS* construct in the Cd-sensitive yeast strain YK44 supported cell growth when the yeast was challenged by 100 µM CdCl_2_ (Fig. 1).

### Transformation of tall fescue with *PaPCS* or *PaGCS* or *PaPCS-PaGCS*

**Figure 2:**
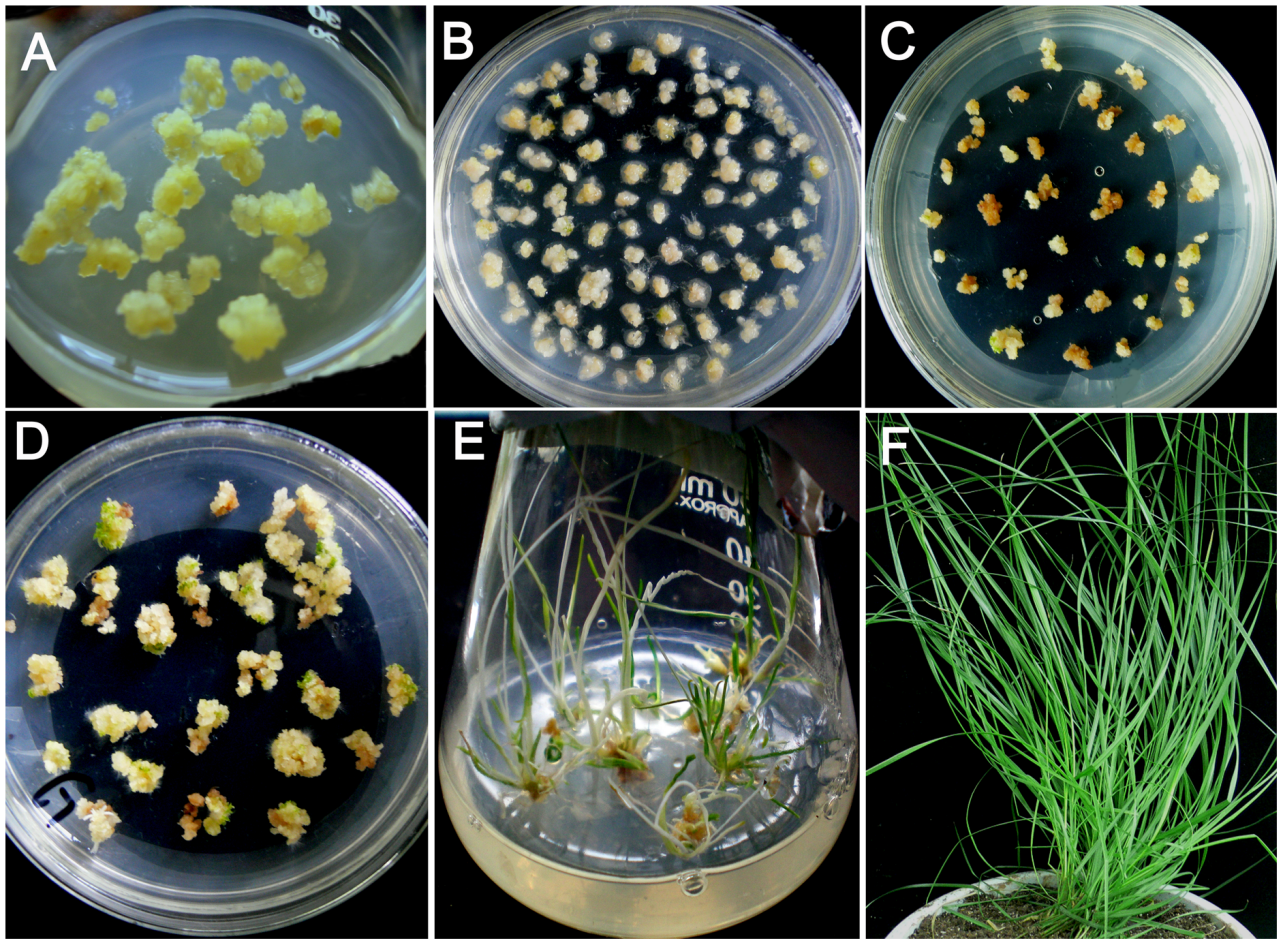
Transformation of *F. arundinacea with PaGCS*. (A) Callus induction, (B) Agroinfection, (C) Kanamycin selection of transgenic calli, (D) Regenerated plants, (E) Selected plants maintain their greenness in the presence of kanamycin, (F) Putative transgenic plants grown in soil.

**Figure 3:**
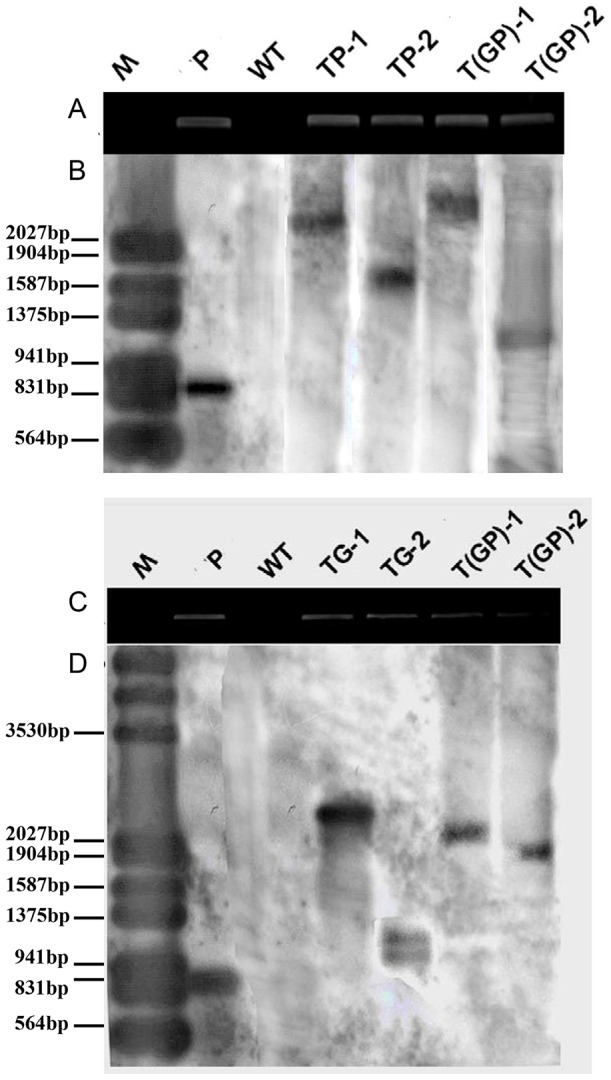
DNA fingerprinting to confirm the presence of *PaPCS* (A,B) and *PaGCS* (C,D). (A,C) PCR analysis, (B,D) Southern blot hybridization of *Eco*RI and *Sac*I digested genomic DNA. TP-1, TP-2: carriers of *PaPCS*; TG-1, TG-2: carriers of *PaGCS*; T(GP)-1, T(GP)-2: carriers of *PaGCS-PaPCS*; P: plasmid; WT: wild type *F. arundinacea*. M: size marker.

Granular embryogenic calli were used for agroinfection (Figs. 2A, S3A, S4A). Two weeks after agroinfection with either pROK/PaGCS, pROK/PaPCS or p3301/PG (Figs. 2B, S3B, S4B), kanamycin resistant calli having a yellow granulated structure were selected (Figs. 2C, S3C, S4C), shoots and roots were induced (Figs. 2D, S3D, S4D) and a second round of kanamycin selection was applied to remove escapes (Figs. 2E, S3E, S4E). In all, 95 putative transgenic *PaPCS* plants, 87 *PaGCS* plants and 15 *PaPCS*/*PaGCS* plants were regenerated (Figs. 2F, S3F, S4F). The PCR-based screening of the putative transgenics identified seven *PaPCS*, eleven *PaGCS* transgenic lines and three *PaPCS*/*PaGCS* transgenic lines. Of these, two per transgene were taken forward. A semi-quantitative RT-PCR analysis showed that transgene transcript appeared in transgenic lines (Fig. 3A,C). Profiling the genomic DNA of the six selected transformants by Southern hybridization showed that a single copy of *PaPCS* was integrated into each of the two *PaPCS* lines, a single copy of *PaGCS* into one of the *PaGCS* lines (and two copies into the other), and a single copy of *PaPCS-PaGCS* into each of the *PaPCS*/*PaGCS* lines (Fig. 3B,D).

### Growth and Cd accumulation of WT and transgenic tall fescue plants

**Figure 4:**
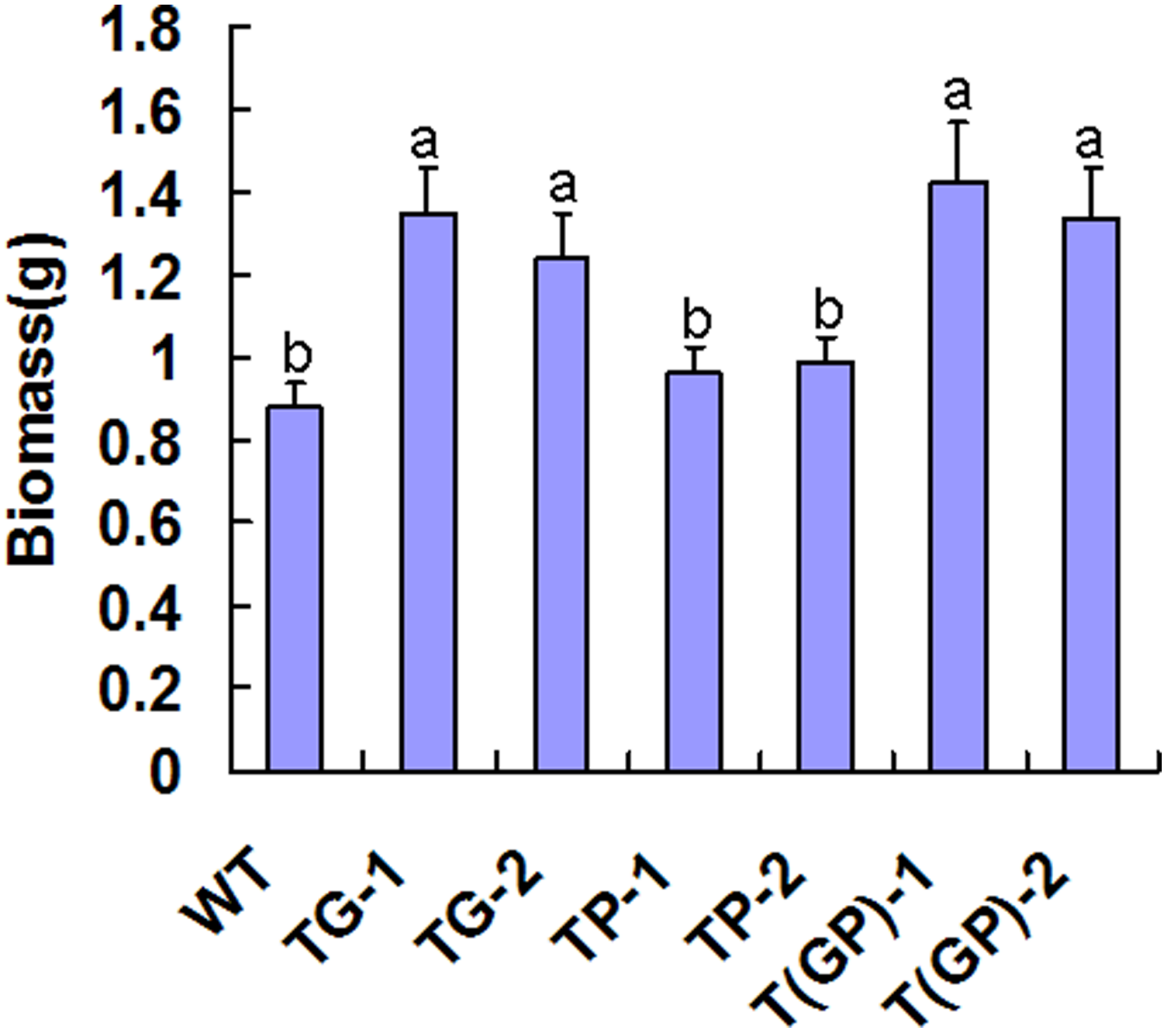
The total biomass accumulation after exposure to 150 µM CdCl_2_ for five days. TP-1, TP-2: carriers of *PaPCS*; TG-1, TG-2: carriers of *PaGCS*; T(GP)-1, T(GP)-2: carriers of *PaGCS-PaPCS*; WT: wild type *F. arundinacea*. Vertical bars indicate the standard error of the mean (n = 4). Different letters indicate significant differences according to a Tukey test (P<0.01).

**Figure 5:**
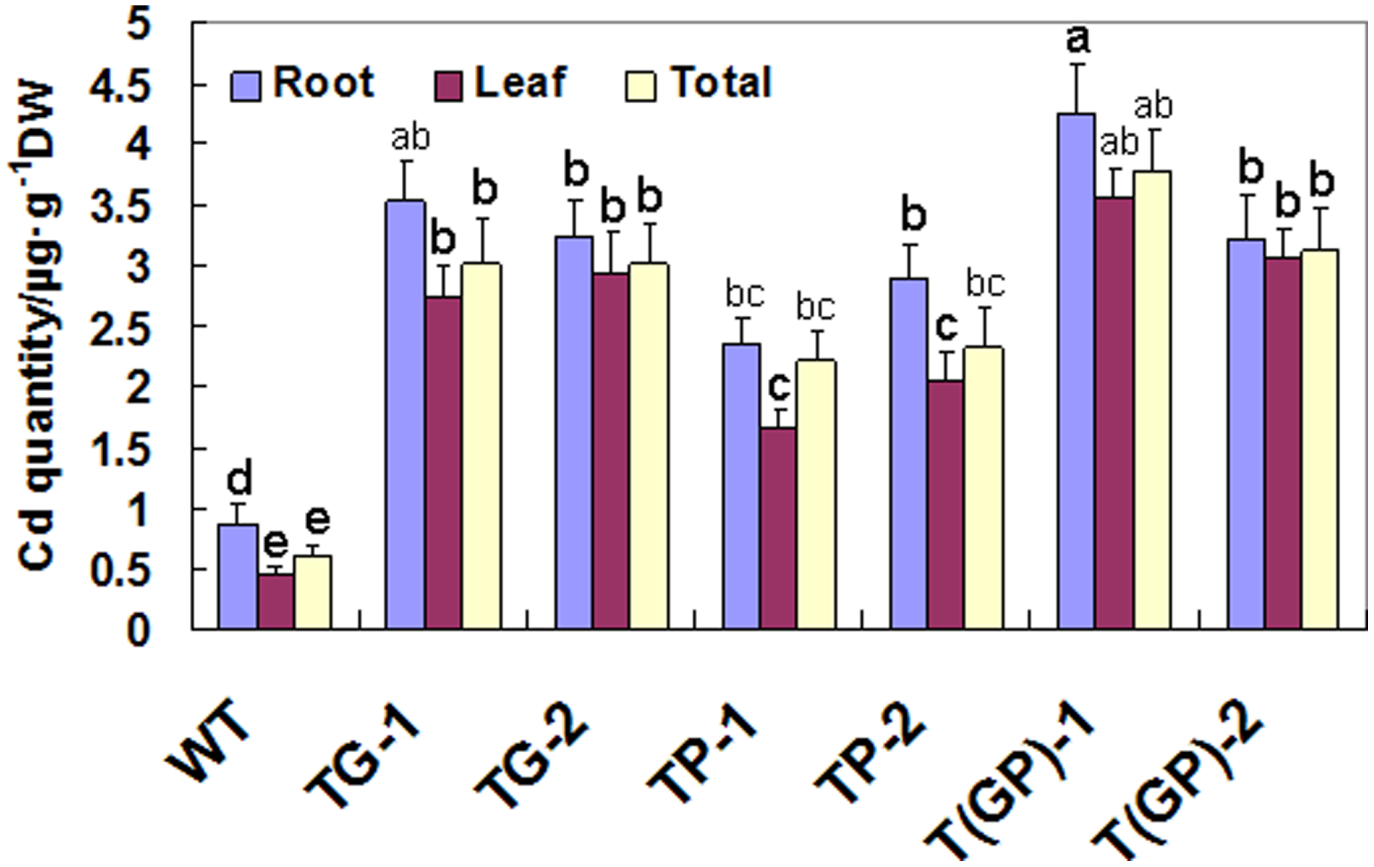
Cd^2+^ accumulation of transgenic lines and wild type after exposure to 150 µM CdCl_2_ for five days. The Cd content of the roots, leaves and total plant was measured in four plants per line. TP-1, TP-2: carriers of *PaPCS*; TG-1, TG-2: carriers of *PaGCS*; T(GP)-1, T(GP)-2: carriers of *PaGCS-PaPCS*; WT: wild type *F. arundinacea*. Vertical bars indicate the standard error of the mean(*n* = 4). Different letters indicate significant differences according to a Tukey test (P<0.01).

After five days exposure to 150 µM CdCl_2_, the total biomass of each of the three types of transgenic lines was greater than that of the WT, with the double transformant being the most productive, suggesting a better Cd tolerance. However, the total biomass increase for *PaPCS* transgenic lines was not significant (Fig. 4). The Cd content of each of the three types of transgenic lines was significant greater than that of the WT in leaf and root, in the order of double transformants>*PaGCS* single transformants>*PaPCS* single transformants>WT (Fig. 5). The root was higher than that of the leaf in each case, and the double transformant accumulated more Cd than did either of the single transformants. The ratio between the leaf and root Cd content was higher for the transgenic material than for the WT. In WT, the Cd content in the root was 1.9 fold higher than that of in the leaf, this ratio was significant decreased in *PaPCS* transgenic lines (1.4 fold) and *PaGCS* transgenic lines (1.1–1.28 fold). In the double transformant, the ratio was 1.0–1.2 fold, which was not significantly different with *PaGCS* transgenic lines (Tab. 1), suggesting these transgenic plant was particularly efficient in translocating Cd from the root to the leaf.

**Table 1:** Ratio of Cd root/leaf content.

plant lines	Ratio of Cd root/leaf content
WT	1.9312±0.165 a
TG-1	1.2878±0.103 c
TG-2	1.1059±0.111 c
TP-1	1.4341±0.121 b
TP-2	1.4058±0.134 b
T(GP)-1	1.1948±0.128 c
T(GP)-2	1.0522±0.110 c

Values are mean ± standard error (n = 4). For each variable, values followed by different letters differ significantly between the strains (Student's t-test, P<0.05).

### MDA content and enzyme activities under Cd stress

**Figure 6:**
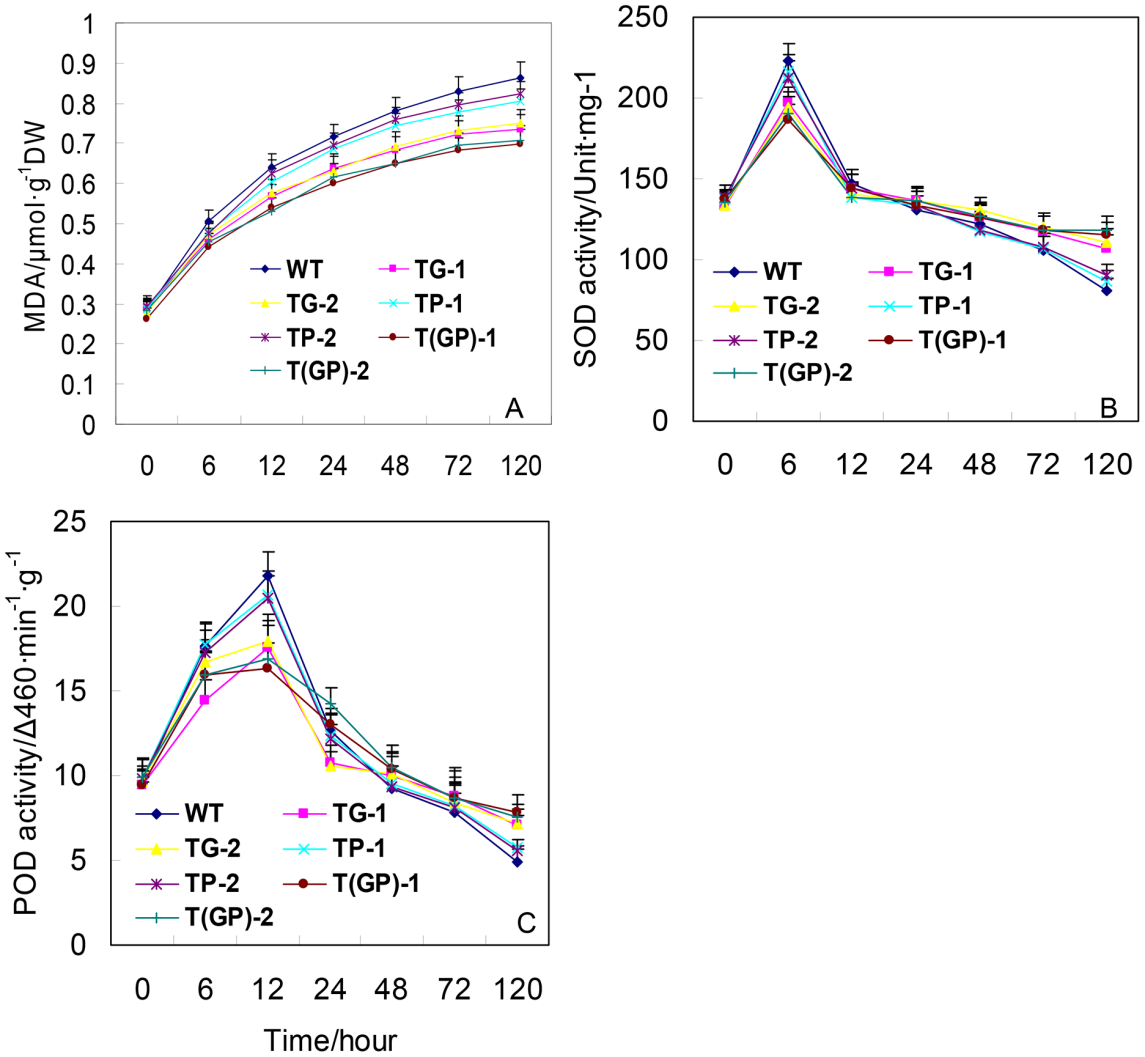
Short term response of plants to Cd exposure/After 0–120 h, the leaves were sampled for (A) MDA content, (B) SOD activity, (C) POD activity. TP-1, TP-2: carriers of *PaPCS*; TG-1, TG-2: carriers of *PaGCS*; T(GP)-1, T(GP)-2: carriers of *PaGCS-PaPCS*; WT: wild type *F. arundinacea*. Vertical bars indicate the standard error of the mean (*n* = 4).

After 6 h of Cd stress, the MDA content of both the transgenic and WT plants began to rise, with the rate of increase being fastest in the WT plants. The rate of increase in MDA content was slowest in the two double transformants, followed first by the *PaGCS* single transformants and then by the *PaPCS* single transformants (Fig. 6A). Thus in the transgenic plants, lipid peroxidation was slowed, and this slowing was more effective in the presence of *PaGCS*.

After 6 h of Cd stress, the MDA content of both the transgenic and WT plants began to rise, with the rate of increase being fastest in the WT plants. The rate of increase in MDA content was slowest in the two double transformants and *PaGCS* single transformants, followed by the *PaPCS* single transformants (Fig. 6A).

Thus in the transgenic plants, lipid peroxidation was slowed, and this slowing was more effective in the presence of *PaGCS*. SOD and POD activity all peaked rather quickly in response to the Cd stress, the former by 6 h, and the latter by 12 h (Figs. 6B, C). At its peak, SOD activity was highest in the WT, followed by the *PaPCS* transgenics, the *PaGCS* transgenics and finally the double transformants (Fig. 6B). At its peak, POD activity had the same pattern as SOD activity (Fig. 6C). After peaking, both SOD and POD activity sharply decreased. Thus, after 24 h of Cd stress, both SOD and POD activity had dropped below their initial level. At this time, the ranking of enzyme activity was *PaGCS*/*PaPCS* transgenics>*PaGCS* transgenics>*PaPCS* transgenics>WT.

### GSH, PC and TNP-SH content of transgenic tall fescue under cadmium stress

**Figure 7:**
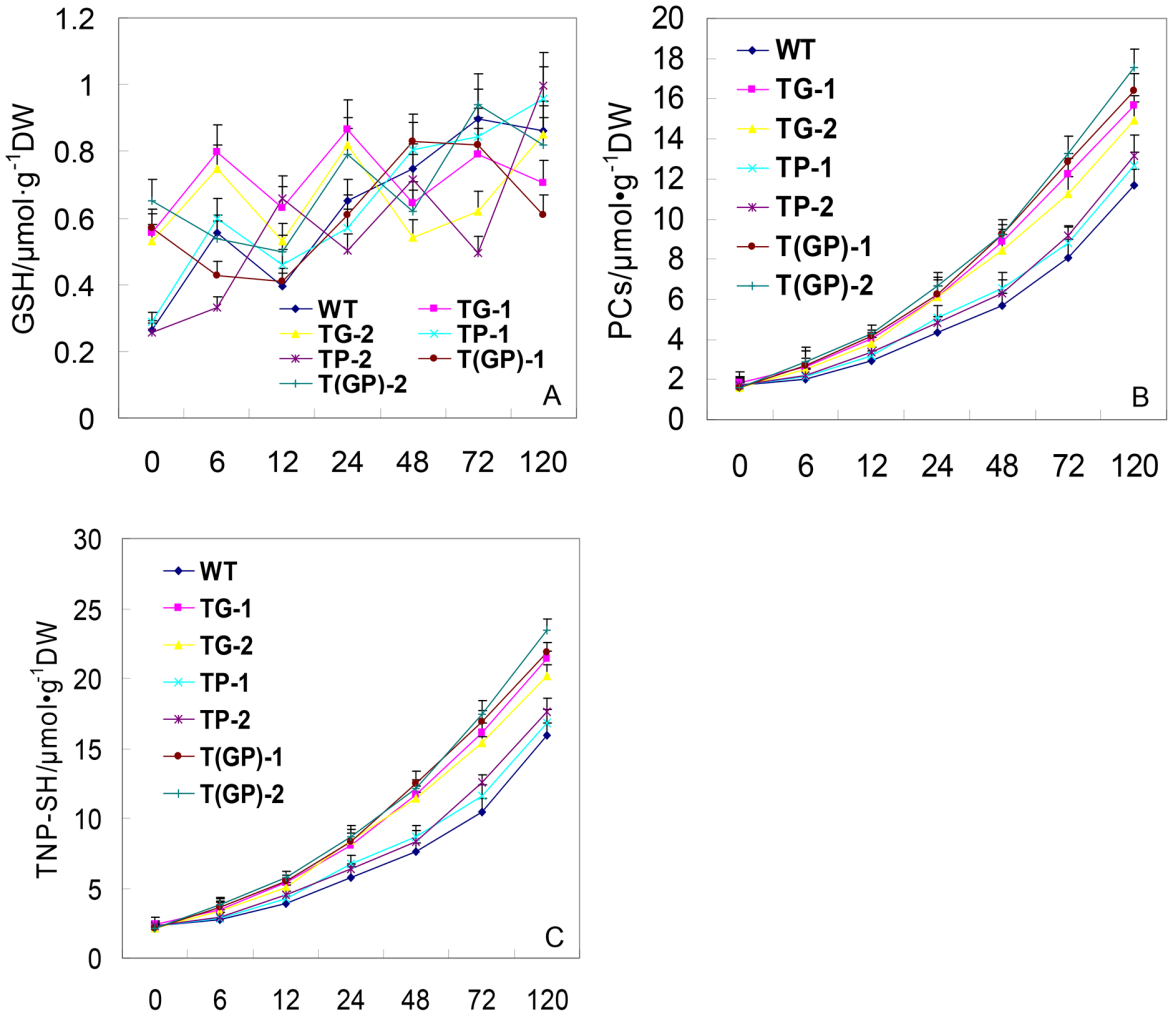
Short term response of plants to Cd exposure/After 0–120 h, the leaves were sampled for (A) Total glutathione (GSH) content, (B) Non-protein thiol (TNP-SH) content, (C) PC content. TP-1, TP-2: carriers of *PaPCS*; TG-1, TG-2: carriers of *PaGCS*; T(GP)-1, T(GP)-2: carriers of *PaGCS-PaPCS*; WT: wild type *F. arundinacea*. Vertical bars indicate the standard error of the mean (*n* = 4).

Before exposure to Cd stress, the total GSH content of the three transgenic types was similar to that of WT plants, as well as the PC and TNP-SH content. As the Cd stress was applied, the total GSH content of WT and the transgenic plants did not significantly change (Fig. 7A), but both the PC and TNP-SH content increased (Figs. 7B, C, S5). Overall, with respect to PC, the double transformants out-performed each of the single transformants; the *PaGCS* transgenic was superior to the *PaPCS* transgenic, and both single transformants out-performed WT (Fig. 7C).

## Discussion

PCS is the enzyme which catalyses the final step in the synthesis of phytochelatin, so it has been the major focus in the field of the genetic engineering of phytoremediation ever since it was first identified by Vatamaniuk et al. [36] and Clemens et al. [37]. Expression of PCS in both prokaryotes and yeast increases tolerance to heavy metals [38]–[40], as well as to various abiotic stresses in *Anabaena* [41]. In the present study, when the *PaPCS* gene was expressed in a strain of yeast sensitive to Cd, the tolerance of the cells to the presence of 100 µM CdCl_2_ in the growth medium was noticeably improved. The introduction of *PCS* genes from various species into plants has also generally resulted in an improved ability to tolerate and/or accumulate heavy metals. Examples include the heterologous expression of *A. thaliana AtPCS1* in tobacco [42] and Indian mustard [43], bread wheat *TaPCS1* and *Ceratophyllum demersum CdPCS1* in tobacco [40], and that of *Nelumbo nucifera NnPCS* and *CdPCS1* in *A. thaliana* [44]–[46]. When a PCS deficient *A. thaliana* mutant was transformed with *AtPCS1* driven by a leaf-specific promoter, the resulting lines proved less sensitive to Cd toxicity [47]. However, although over-expression of *AtPCS1* helped raise the level of tolerance to arsenic [48], it also resulted in a higher sensitivity to Cd [49]. In the present study heterologous expression of *PaPCS* in tall fescue improved the plants' ability to accumulate biomass in the presence of 150 µM CdCl_2_, which demonstrated the contribution that this gene makes to Cd tolerance.

However, the previous studies paid more attention on single overexpression of genes involved in chelation, and it indicated that overexpression of genes involved in PCs synthesis in plants results in either hypersensitivity or tolerance to Cd [50]–[51]. In our study, the line overexpressed single PaPCS or PaGCS, was tolerant to Cd stress (Fig. 4). It had higher content of PCs (Fig. 7) and accumulated higher amounts of this metal (Fig. 5) than WT. This result supported some of the above reports. In our study, the transgenic lines simultaneous overexpressed PaPCS and PaGCS gene had higher contents of Cd and PCs than WT and single-gene transgenic lines after Cd exposure (Fig. 5,7). This result is consistent with the previous study on the transgenic lines simultaneous overexpressed CsPCS and YCF1 which accumulated higher amounts of Cd than single-gene transformants [52]. This showed that multiple overexpression of genes involved in chelation can led to a higher increase on the tolerance and accumulation to heavy metal, it might be a way of acquiring more efficient hyperaccumulator.

The behaviour of MDA content and the patterns of SOD and POD activity confirmed this conclusion, since the leaf content of MDA in the non-transgenic plants remained substantially higher than in the *PaPCS* transgenic lines (Fig. 6A). The significance of MDA content lies in its being a diagnostic for the severity of oxidative stress. It is suggested that the level of oxidative damage in the transgenic lines was less severe than in the WT. Consistently, both SOD and POD activity were higher in the *PaPCS* transgenics than in the WT 24 h after the stress was imposed (Fig. 6B, 6C). Heavy metals in their ionic state are considered to be rather more toxic than when they are complexed with organic molecules, for instance the PCs. The *PaPCS* transgenic lines accumulated more Cd than the WT, but at the same time they were able to neutralize this additional Cd by enhancing the cellular level of PC.

It has been noted that the tissue-specific expression of PC biosynthesis genes can result in the relocation of heavy metal mediated by the long distance xylem transport of γ-glutamylcysteine, and PCs [53], [54]. However, little is known about the relationship between growth condition, PC content and redistribution of Cd of the transgenic lines with single or double PC biosynthesis genes. Here, we detected the variation of the redistribution of Cd of the WT and three transgenic lines, and found that the heterologous overexpression of *PaGCS* in tall fescue increased the ratio of Cd root/leaf content (Tab. 1), which is consistent with the previous study of *PaGCS* heterologous overexpression in *Agrostis palustris* [26]. Heterologous overexpression of *PaPCS* in tall fescue also increased the ratio of Cd root/leaf content (Table 1). This phenomenon has been observed in previous study of wheat PCS overexpressed in rice, in which the Cd accumulation were increased in shoot, but not changed in root [55]. However, other study did not show the redistribution of Cd in PCS transgenic plants, which suggested the redistribution might depend on the gene sources [52], [53], [56].

In previous study, the growth of *PCS* transgenic plants appeared to be inhibited under Cd stress, although when the plants were given exogenous GSH, this sensitivity disappeared. The interpretation of this behavior was that glutathione must be necessary for the operation of PCS. γ-GCS catalyzes the initial step of GSH synthesis, and it is generally understood that GSH synthesis is a rate limiting step for PCS activity. Heterologous expression of the *E. coli* γ-GCS gene *gshl* in poplar chloroplasts is known to enhance the accumulation of GSH [56]. When *PaGCS* and *PaPCS* were simultaneously introduced into tall fescue, the double transformant line out-performed both of the single transgene carriers, which were superior to the WT. This out-performance was measurable in terms of biomass accumulation, MDA content and antioxidant enzyme activity. The implication is that of the two genes *PaGCS* and *PaPCS*, the former has a stronger beneficial effect than the latter on heavy metal tolerance.

## Supporting Information

Figure S1 **The plant expression vectors (A) pROK/PaGCS, and (B) p3301/PG. RB and LB: right and left borders of the** ***Ti*** **plasmid;** ***Tnos*** **: nos terminator (260 bp).**
(TIF)

Figure S2 **Phylogeny of deduced phytochelatin synthase polypeptides.**
(TIF)

Figure S3 **Transformation of F. arundinacea** ***with PaPCS*** **.** (A) Callus induction, (B) Agroinfection, (C) Kanamycin selection of transgenic calli, (D) Regenerated plants, (E) Selected plants maintain their greenness in the presence of kanamycin, (F) Putative transgenic plants grown in soil.
(TIF)

Figure S4 **Transformation of** ***F. arundinacea*** **with** ***PaGCS-PaPCS*** **.** (A) Callus induction, (B) Agroinfection, (C) Kanamycin selection of transgenic calli, (D) Regenerated plants, (E) Selected plants maintain their greenness in the presence of kanamycin, (F) Putative transgenic plants grown in soil.
(TIF)

Figure S5 **PC2 and PC3 content after short term Cd exposure/After 0–120 h, the leaves were sampled for (A) PC2 and (B) PC3. TP-1, TP-2: carriers of** ***PaPCS*** **; TG-1, TG-2: carriers of** ***PaGCS*** **; T(GP)-1, T(GP)-2: carriers of** ***PaGCS-PaPCS*** **; WT: wild type** ***F. arundinacea*** **.** Vertical bars indicate the standard error of the mean (*n* = 4).
(TIF)
