# Supplementary figures and images for: Cloning and Characterization of a Phragmites australis Phytochelatin Synthase (PaPCS) and Achieving Cd Tolerance in Tall Fescue
Source: PLoS One. 2014 Aug 18;9(8):e103771. doi: 10.1371/journal.pone.0103771 (PMC4136729; doi:10.1371/journal.pone.0103771)

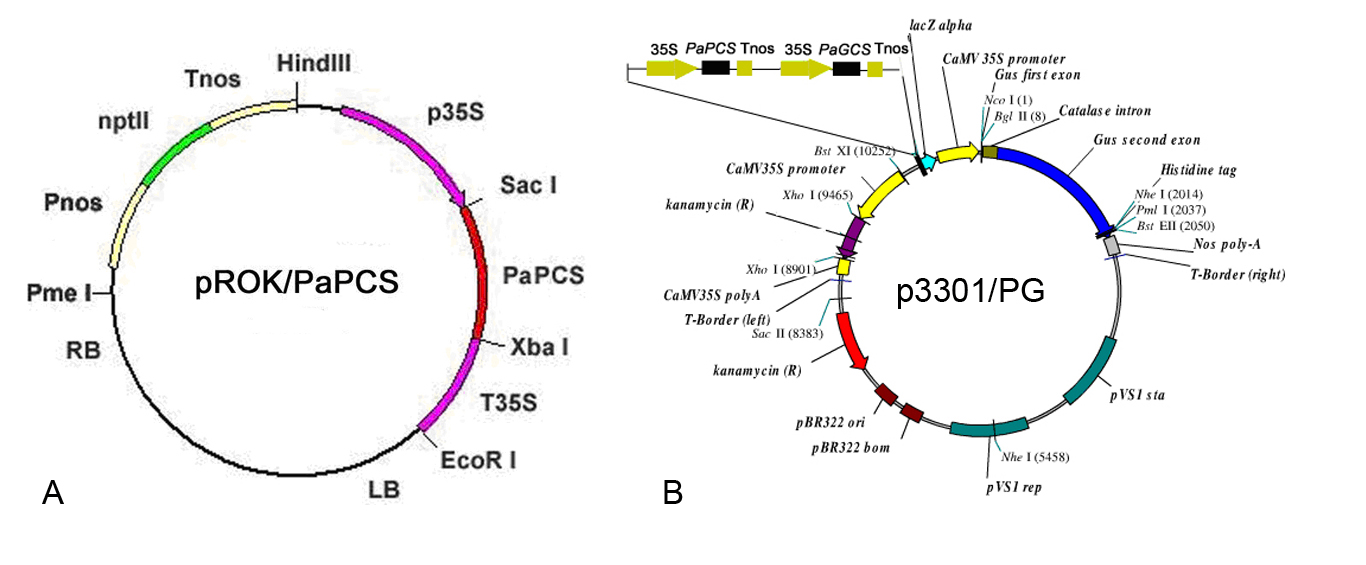

Supplement: Figure S1 — The plant expression vectors (A) pROK/PaGCS, and (B) p3301/PG. RB and LB: right and left borders of the Ti plasmid; Tnos : nos terminator (260 bp). (TIF) [file pone.0103771.s001.tif]

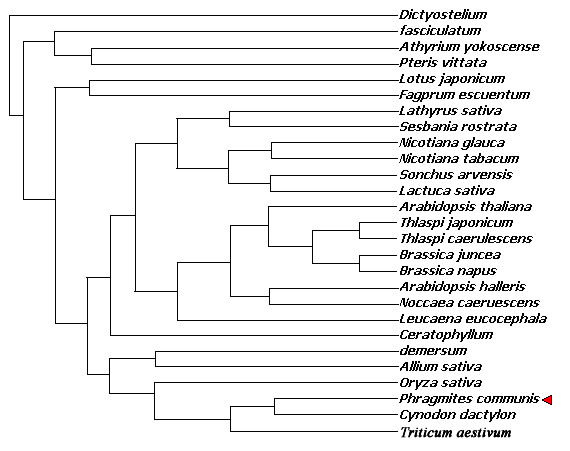

Supplement: Figure S2 — Phylogeny of deduced phytochelatin synthase polypeptides. (TIF) [file pone.0103771.s002.tif]

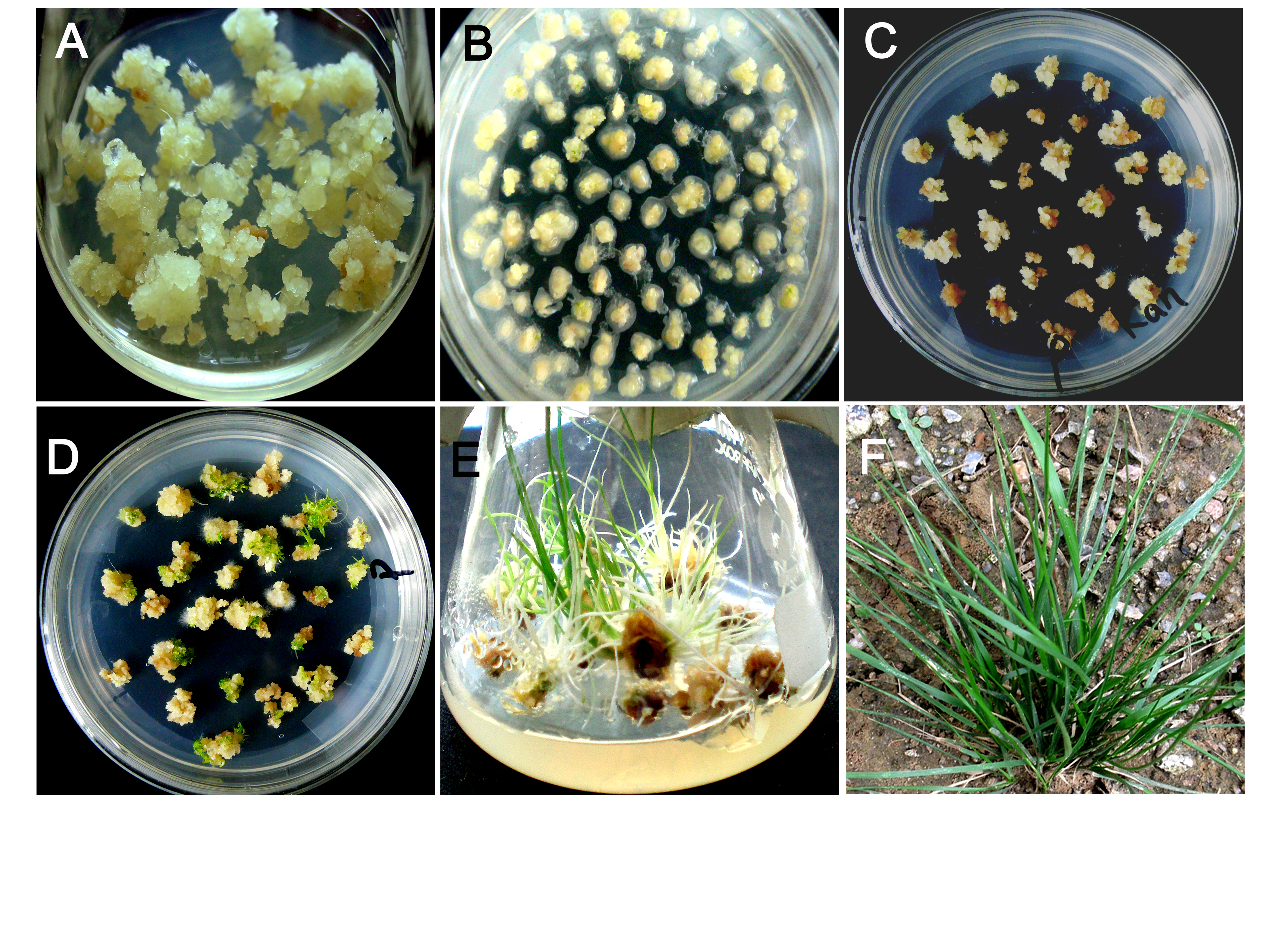

Supplement: Figure S3 — Transformation of F. arundinacea with PaPCS . (A) Callus induction, (B) Agroinfection, (C) Kanamycin selection of transgenic calli, (D) Regenerated plants, (E) Selected plants maintain their greenness in the presence of kanamycin, (F) Putative transgenic plants grown in soil. (TIF) [file pone.0103771.s003.tif]

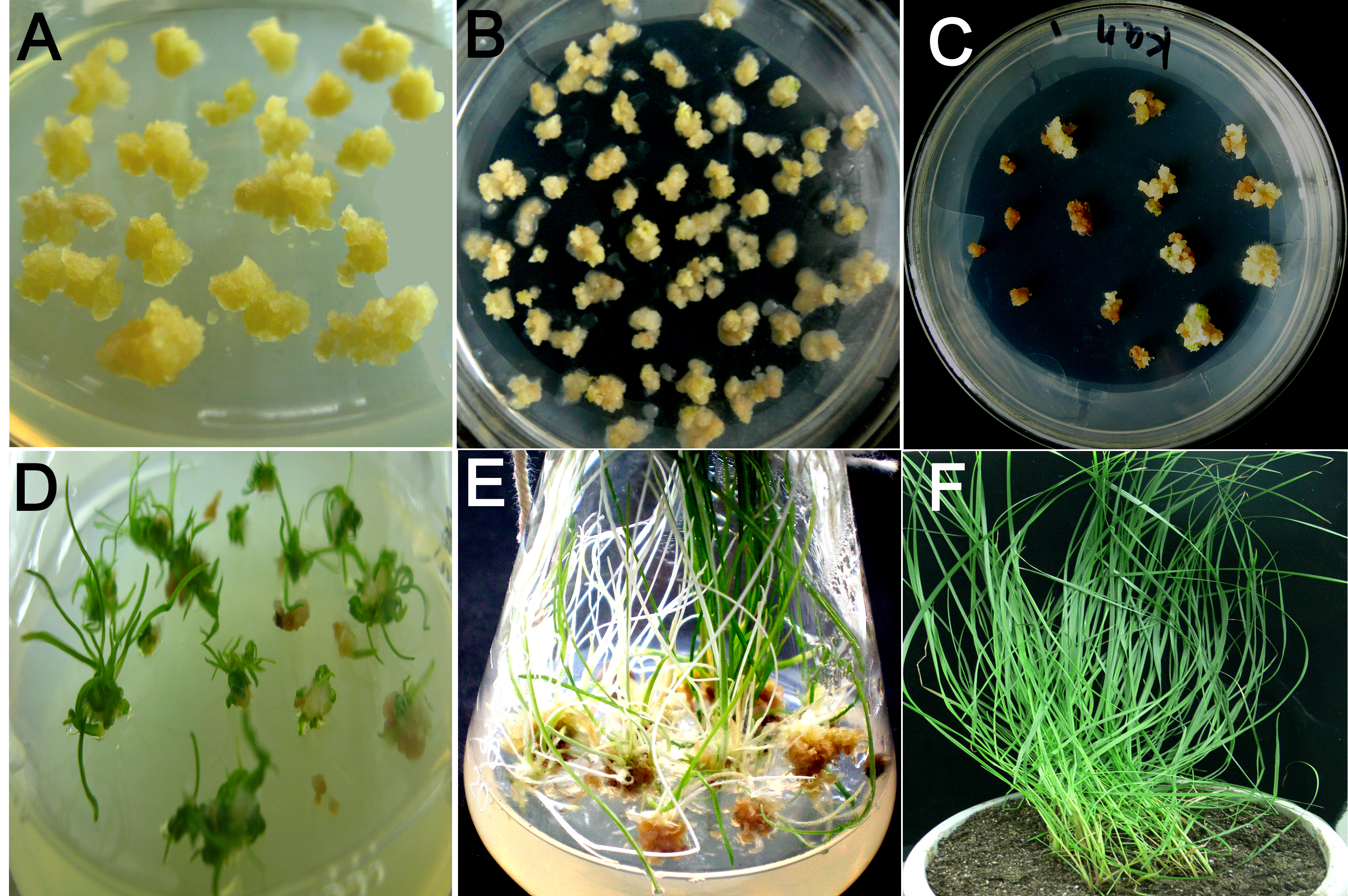

Supplement: Figure S4 — Transformation of F. arundinacea with PaGCS-PaPCS . (A) Callus induction, (B) Agroinfection, (C) Kanamycin selection of transgenic calli, (D) Regenerated plants, (E) Selected plants maintain their greenness in the presence of kanamycin, (F) Putative transgenic plants grown in soil. (TIF) [file pone.0103771.s004.tif]

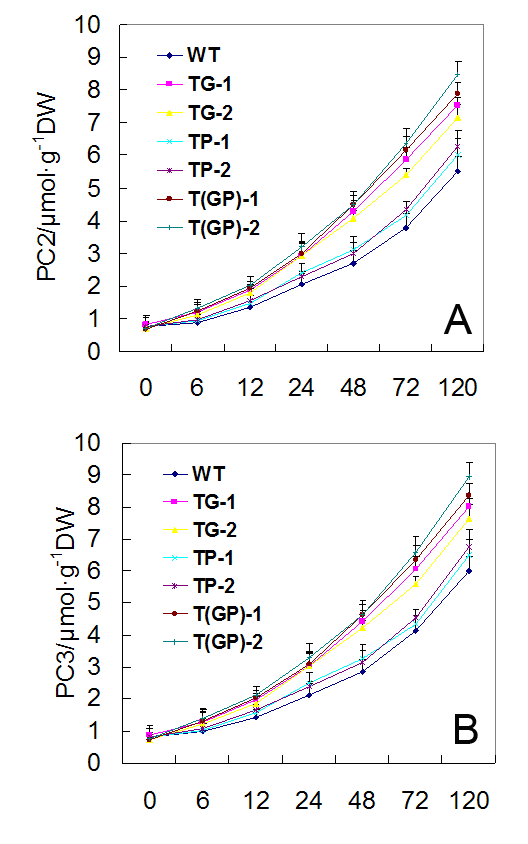

Supplement: Figure S5 — PC2 and PC3 content after short term Cd exposure/After 0–120 h, the leaves were sampled for (A) PC2 and (B) PC3. TP-1, TP-2: carriers of PaPCS ; TG-1, TG-2: carriers of PaGCS ; T(GP)-1, T(GP)-2: carriers of PaGCS-PaPCS ; WT: wild type F. arundinacea . Vertical bars indicate the standard error of the mean (n = 4). (TIF) [file pone.0103771.s005.tif]
